# Supplementary material for: Proximity-induced magnetism in transition-metal substituted graphene
Source: Sci Rep. 2015 Aug 3;5:12322. doi: 10.1038/srep12322 (PMC4522683; doi:10.1038/srep12322)
Supplement: Supplementary Information [file srep12322-s1.pdf]

Proximity-induced magnetism in transition-metal substituted graphene: Supplemental material

Charles B. Crook[1], Costel Constantin[1], Towfiq Ahmed[2], Jian-Xin Zhu[2,3], Alexander V. Balatsky[4,5], and Jason T. Haraldsen[1]
[1]*Department of Physics and Astronomy, James Madison University, Harrisonburg, VA 22802*
[2]*Theoretical Division, Los Alamos National Laboratory, Los Alamos, NM 87545, USA*
[3]*Center for Integrated Nanotechnologies, Los Alamos National Laboratory, Los Alamos, NM 87545, USA*
[4]*Institute for Materials Science, Los Alamos National Laboratory, Los Alamos, NM 87545, USA and*
[5]*NORDITA, Roslagstullsbacken 23, 106 91 Stockholm, Sweden*

In this supplementary material section, we examine and present the data relative to the distorted graphene sheet and density of states, and we provide details into the effects of an added Hubbard U. The first graph introduces the change in bond length and bond angle in the six configurations used with each magnetic atom, vanadium, chromium, and manganese. Whilst the second graph displays the calculated total density of state in graphene among the six arrangements between the two magnetic atoms. We also show comparisons to the calculations with an onsite Hubbard U potential to show that there is no change in the overall conclusions of the paper.

UNDERSTANDING THE DISTORTION

As show in Fig. S1, we present the bond lengths and bond angles of the six separations using three different magnetic atoms, vanadium, chromium, and manganese. In Fig. S1(a), we see the average bond lengths over the six configurations. The vanadium-substituted graphene seems to be near $1.85\AA$, the chromium around $1.95\AA$, and the manganese at $1.80\AA$. We can see that these have increased from the average C-C bond length of $1.42\AA$.

In Fig. S1(b), the average bond angles are now compared of the C-M-C bonds. As the number of carbon atoms between the two magnetic atoms increase, the bond angle decreases with all three. The vanadium-substituted seems to level off at 92°, while the chromium and manganese substituted seems to approach 88° and 94° respectively. Compared to the regular C-C-C bond angle in a plain graphene sheet, all three of these have decreased from 120°.

DENSITY OF STATES

Fig. S2 displays the total density of state of the carbon atoms between the two magnetic atoms in the six separations of the three possible magnetic atoms. The number of carbon atoms separated are 1(a), 2(b), and onwards till 6(f). The density of states of Dirac materials creates a gap in the presence of impurities, which is observed around -1 to 1 eV. However, the presence of impurities produce electronic bands at the Fermi level, which allows for the increased conductance. This is a well known feature of Dirac materials[1].

COMPARISON TO CALCULATIONS WITH AN ONSITE POTENTIAL

In manuscript, we refer to the fact that we are performing these calculations without an onsite potential or Hubbard U value. This is due to two main reasons: 1) The use of an onsite potential would increase computational time, and 2) the value of the onsite potential is typically determined through the comparison of experimental data. We ran a few test cases for the magnetic atoms in graphene to illustrate that the presence of an onsite potential does not great effect the overall structure or underlying findings. Figure S3 shows the geometrically minimized structure configuration with and without an onsite potential (U = 4.0 eV on the 3d electrons) for 4 carbon separated Mn-substituted graphene. The bottom plots are the magnetic moment for each atom both with and without the U value for the same Mn case.

This demonstrates that the presence of a Hubbard U potential only affects the structure by about 5%, while still allowing for the magnetic structure to remain. In Figure S4, we show the exchange energy as a function of carbon separation for the Mn cases. Here, the onsite potential does change the overall ground state for the magnetic atoms, but the overall oscillation of the exchange energy remains. While the distance dependence is dampened due to the large U value, the oscillatory nature remains. This is due to the presence of the onsite potential shifting the overall bandstructure. However, the increase the Fermi level resonance in the density of states is still present due to impurity-band scattering, which is a fundamental feature of Dirac materials[1].

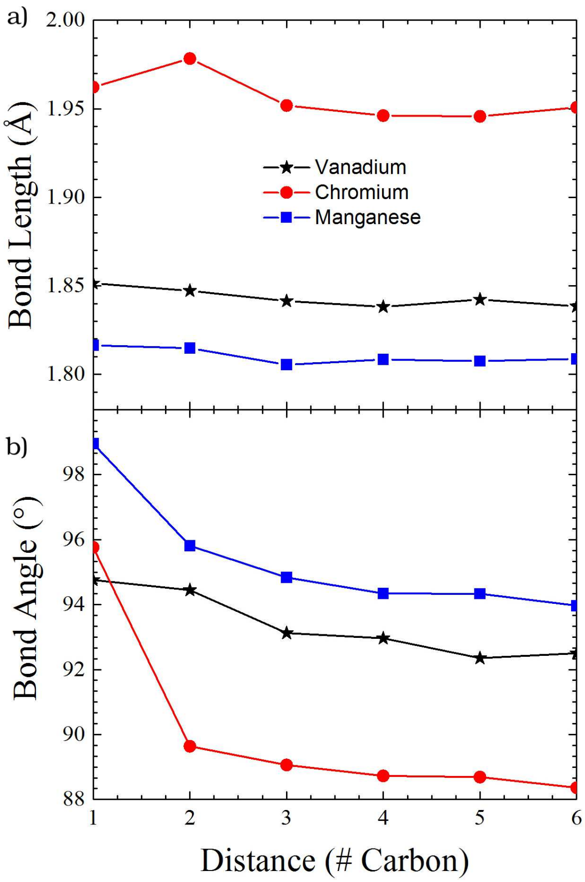

FIG. S1: a) Calculated average bond length between magnetic atom and carbon for vanadium (black stars), chromium (red circles), and manganese (blue squares) substituted graphene. b) Calculated average bond angle two carbons connecting to an atom in graphene.

Overall, this substantiates that while an onsite Hubbard U potential produces slightly different results, the main points demonstrated in this manuscript still hold true. This includes: proximity induced magnetism in graphene, Fermi level resonance, and oscillatory exchange interactions that are characteristic of an RKKY interaction. However, since only experiments can be used to determine the correct amount of onsite potential needed, it is hoped that this work will motivate research activity into the area of direct magnetic substitution in 2D materials.

[1] Wehling, T. O., Black-Schaffer, A. M. & Balatsky, A. V. Dirac materials. *Advances in Physics* **63**, 1-76 (2014).

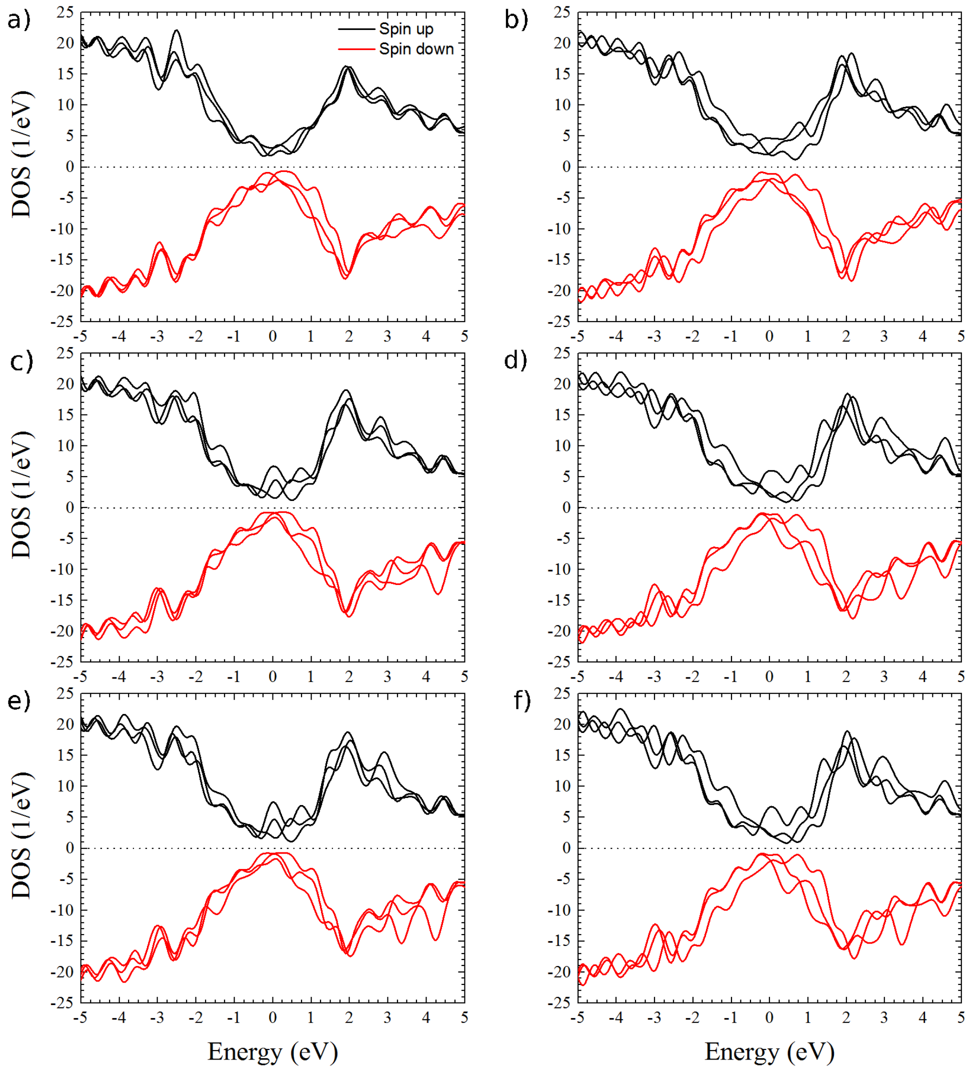

FIG. S2: The calculated total density of state in graphene with odd (1(a), 3(c), 5(e)) and even (2(b), 4(d), 6(f)) separations between the two magnetic atoms for each configuration.

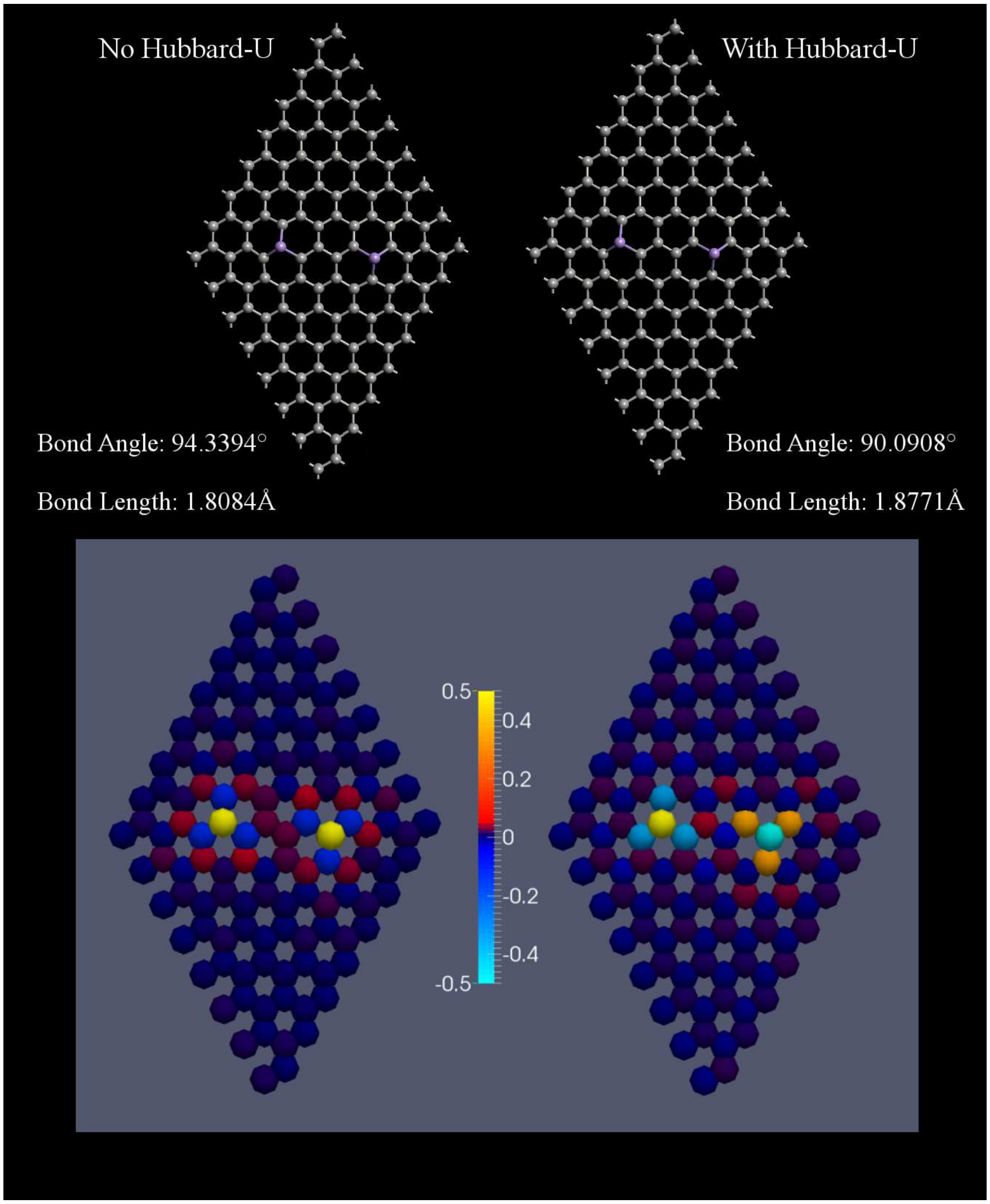

FIG. S3: Comparison of the structure and magnetic moment simulations with and without an onsite Hubbard U potential the 4 carbon separated Mn-substituted graphene.

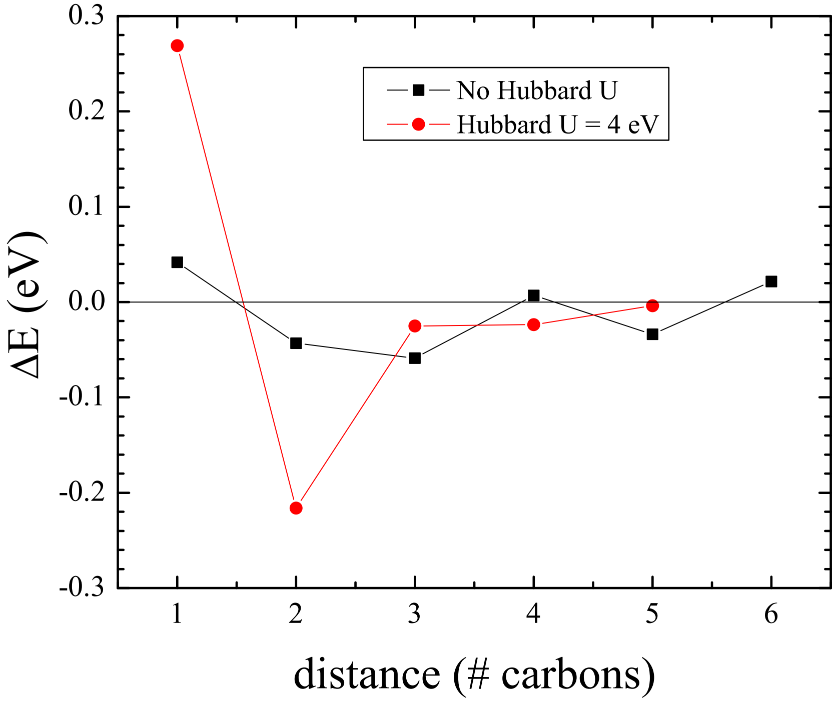

FIG. S4: Comparison of the exchange interactions as a function of magnetic atom separation (in number of carbons) for Mn-substituted graphene.
